# An overview of salinity stress, mechanism of salinity tolerance and strategies for its management in cotton

**DOI:** 10.3389/fpls.2022.907937

**Published:** 2022-10-07

**Authors:** Zahra Maryum, Tahira Luqman, Sahar Nadeem, Sana Muhy Ud Din Khan, Baohua Wang, Allah Ditta, Muhammad Kashif Riaz Khan

**Affiliations:** ^1^ Nuclear Institute for Agriculture and Biology-Constituent College (NIAB-C), Pakistan Institute of Engineering and Applied Science Nilore, Islamabad, Pakistan; ^2^ Plant Breeding and Genetics Division, Cotton Group, Nuclear Institute for Agriculture and Biology, Faisalabad, Pakistan; ^3^ School of Life Sciences, Nantong University, Nantong, China

**Keywords:** *Gossypium*, cotton, salinity stress, salinity tolerance, marker assisted selection, genomics

## Abstract

Salinity stress is one of the primary threats to agricultural crops resulting in impaired crop growth and development. Although cotton is considered as reasonably salt tolerant, it is sensitive to salt stress at some critical stages like germination, flowering, boll formation, resulting in reduced biomass and fiber production. The mechanism of partial ion exclusion (exclusion of Na^+^ and/or Cl^–^) in cotton appears to be responsible for the pattern of uptake and accumulation of harmful ions (Na^+^ and Cl) in tissues of plants exposed to saline conditions. Maintaining high tissue K^+^/Na^+^ and Ca^2+^/Na^+^ ratios has been proposed as a key selection factor for salt tolerance in cotton. The key adaptation mechanism in cotton under salt stress is excessive sodium exclusion or compartmentation. Among the cultivated species of cotton, Egyptian cotton (*Gossypium barbadense* L.) exhibit better salt tolerance with good fiber quality traits as compared to most cultivated cotton and it can be used to improve five quality traits and transfer salt tolerance into Upland or American cotton (*Gossypium hirsutum* L.) by interspecific introgression. Cotton genetic studies on salt tolerance revealed that the majority of growth, yield, and fiber traits are genetically determined, and controlled by quantitative trait loci (QTLs). Molecular markers linked to genes or QTLs affecting key traits have been identified, and they could be utilized as an indirect selection criterion to enhance breeding efficiency through marker-assisted selection (MAS). Transfer of genes for compatible solute, which are an important aspect of ion compartmentation, into salt-sensitive species is, theoretically, a simple strategy to improve tolerance. The expression of particular stress-related genes is involved in plant adaptation to environmental stressors. As a result, enhancing tolerance to salt stress can be achieved by marker assisted selection added with modern gene editing tools can boost the breeding strategies that defend and uphold the structure and function of cellular components. The intent of this review was to recapitulate the advancements in salt screening methods, tolerant germplasm sources and their inheritance, biochemical, morpho-physiological, and molecular characteristics, transgenic approaches, and QTLs for salt tolerance in cotton.

## 1 Introduction

**Figure 1:**
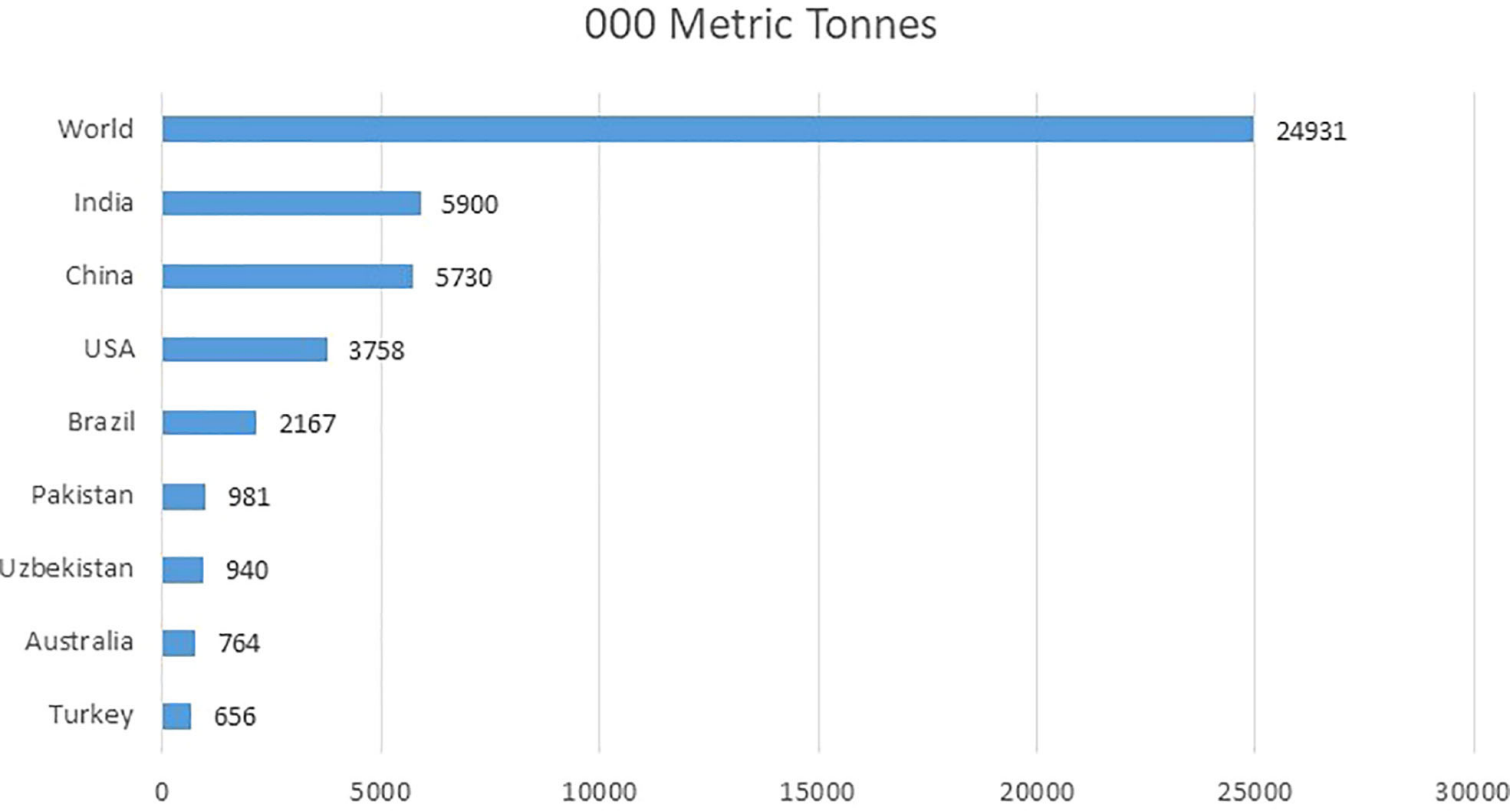
Top cotton producing countries worldwide in 2021-2022 (in 1,000 metric tons) (International Cotton Advisory Committee, 2021-2022).

Cotton (*Gossypium* spp.) is one of the most economically important crop in the world, as it serves as the chief source of natural fiber contributing nearly 35% of the total fiber used in the world. It is also known as “white gold” in some countries due to the huge amount of revenue it is generating (Ali et al., 2014). The top five cotton-producing countries in the world are India, China, the USA, Brazil, and Pakistan (Meyer, 2021). Together these countries contribute almost 2/3 of the world’s cotton. One of the biggest textile industries of the world are extending by cotton’s fiber produce with an annual economic impact of approximately $600 billion worldwide (Ashraf et al., 2018). The total annual production of cotton worldwide is closely 25M tons (Khan et al., 2020). **Figure 1** is showing production of top cotton producing countries in thousand metric tonnes. Pakistan is the world’s 5^th^ largest producer of cotton (International Cotton Advisory Committee, 2021-2022) and the 7^th^ largest manufacturer of cloth. It is the major cash crop of Pakistan. Almost 60% of the overseas earnings of Pakistan are produced by cotton.

Pakistan’s cotton belt lengthens over 1200 km along with the Indus river among the latitudes of 27°N to 35°N and altitudes from 27 m to 155 m. The soil changes from clay loam to sandy with clay dominant towards the south (ADB, 2009).

Cotton is a fine, soft staple fiber that is formed inside a boll or protective case, alongside the seeds of the plant. It belongs to the genus *Gossypium* and the mallow family Malvaceae (Cobley, 1956). *Gossypium* comprises more than 50 species out of which four species are cultivated worldwide i.e. two diploid species (*G. herbaceum* and *G. arboreum*) and two tetraploid species (*G. hirsutum* and *G. barbadense*). However, *G. hirsutum* that is also known as American cotton or upland cotton contributes towards more than 90% of worldwide production of cotton. It is cultivated on 95% of the cotton-growing area in 17 U.S. states from Virginia to California and contributes towards approximately 97% of the USA’s production of cotton (Meyer, 2018). The reasons because American cotton is grown in more than 80 countries of the world, are its greater yield potential and widespread adaptability. *G. barbadense*, which is also called Egyptian cotton or Pima cotton, is known for its extra-long and extra-fine quality staple. Less than 5% of the world’s cotton production comes from *G. barbadense.*

Currently, the cotton which is grown worldwide is the result of severe selection for the production of high quality fibers to improve harvesting and processing. Continuous selection for similar traits has led to some side effects like narrowing the genetic base and diversity of some traits such as disease resistance and drought and salt tolerance (Rosenow et al., 1983). These narrowed-down genetic resources have been exploited over the years, but, a significant decrease in the production of cotton has been observed for the last few decades (Helms, 2000). To overcome this problem, one of the possible solutions is to bring genes from wild progenitors into the cultivated species which would bring basic genetic solutions to different agricultural trials (Gur and Zamir, 2004). Some of the wild progenitors tolerant to various abiotic stresses are *G. tomentosum* and *G. darwinii. G. tomentosum* exhibits great tendency of salt and drought tolerance because of its unique agronomic traits while *G. darwinii* has shown considerable amount of drought tolerance, disease resistance, and fiber fineness (Liu et al., 2015a). Genomic technologies have been utilized widely for enhancing fiber quality and developing new cotton cultivars having tolerance to biotic and abiotic stresses. It has been supported by the fact that genetic manipulation served as a revolution in the development of improved genomes (Abelson, 1998). Over the last few decades, several omics techniques like transcriptomics, proteomics, and metabolomics have been utilized in comprehension of the processes and mechanisms regarding abiotic stress responses of a plant (Soda et al., 2015).

A typical cotton plant’s growth and development show a distinguishing and unique pattern that has been well defined (Kerby et al., 2010). As compared to other major field crops, cotton is known for its very complicated structure because of its unique indeterminate pattern of growth and sympodial flowering habit (Mauney, 1986). However, that indeterminate growth pattern can be divided into different developmental phases that the crop follows throughout its life. These developmental phases of cotton plant are divided into five major growth stages: (i) germination and emergence, (ii) establishment of seedlings, (iii) development of leaf area and canopy, (iv) flowering and boll development, and (v) maturity (Oosterhuis, 1990). After extensive studies, it has been observed that the flowering and boll development phase is the most critical stage as at this time, plant requirements for resources increase at an exponential rate. Hence, at this stage, the plant is more susceptible to poor management and environmental stresses.

Recent UN projections suggest that the world population could rise up to more than two billion people from today’s level, making it 9.15 billion by the year 2050 (Alexandratos and Bruinsma, 2012), and climatic changes are also evident day by day. Crop production worldwide faces major challenges from biotic and abiotic stresses, and rapid climate change is further contributing towards more acute forms of these biotic and abiotic stresses. It is now of vital importance to increase crop production by at least 40% in arid and semi-arid areas, as the demands for food, fiber, clean water, and bioenergy are getting higher day by day (Nakashima et al., 2014; Shaar-Moshe et al., 2017). Thus, crop production should have to be tailored in such a way that leads to the development of crops resistant to harsh climatic conditions including stress tolerant crops, to maintain world agricultural production.

This review focuses on the,

- effect of salinity on the growth and development of cotton,
- how cotton plant response to salinity stress and detail of its defense mechanism, and,
- management strategies to combat the adverse effects of salt stress in cotton including exploration of cotton genome for salt tolerant genes.

## 2. Abiotic stresses in cotton

Abiotic stress is defined as the negative effect of all the non-living factors on the living organisms in a definite environment. These stresses include salinity or salt stress, drought, extreme temperatures (low or high), and other unfavorable environments (Acquaah, 2009). Among these stresses, high salt levels in soil and water stress are one of the major causes of low crop production worldwide. A plant’s genetic response to abiotic stresses is multigenic, so it is more challenging to identify these responses, control and manipulate them. That is the reason why the improvement of crops for abiotic stresses by conventional breeding methods is limited (Bressan et al., 2009).

Cotton, being a glycophyte, is comparatively more tolerant to abiotic stresses as compared to other main crops. Yet, severe environmental conditions like high levels of salinity and water stress can affect its growth, productivity and quality of produce (fiber) (Parida et al., 2007). Drought affects 45% of agricultural land worldwide, while 19.5% out of irrigated lands is considered saline (Flowers and Yeo, 1995; Dos Reis et al., 2012). Drought accompanied by salinity stress is projected to cause up to 50% loss of fertile land in the next 40 years (Wang et al., 2003). Reportedly, no cotton cultivar is available commercially which is considered tolerant to abiotic stress with high production, yield, and fiber quality (Higbie et al., 2010). Although, cotton with a threshold of 7.7 dSm^-1^, is categorized as a moderately salt tolerant crop (Maas and Hoffman, 1977).

## 3. Salinity stress

Soil salinization is one of the most imperative worldwide problems that negatively affect the productivity of agricultural crops. It has adversely affected more than 800 million hectares of cultivable land worldwide which makes up more than 6% of total agricultural land in the world (Roy et al., 2006). Whereas in Pakistan, salinity has affected 6.28 million hectares of area (Commission, 2012).

Increase in salt content of soil leads to salinity and the salts that mainly contribute towards it are sodium chloride (NaCl) and sodium sulphate (Na_2_SO_4_) (Pessarakli and Szabolcs, 2019). This increase in salt concentration could be due to saline or poor quality irrigation water (Hasanuzzaman et al., 2013) or mal-textured soil which has hampered porosity and aeration (Yadav et al., 2011). Also some areas of the world receive inadequate rainfall causing hindrance in efficient leaching of salts from root zone (Francois et al., 1994). However, in Pakistan, one of the main factor causing salinity is the imprudent use brackish underground water as irrigation water (Malik et al., 2021). Worldwide, irrigated agriculture is a major reason for salinity, which frequently leads to secondary salinization of land and water resources in arid and semi-arid conditions.

When the plant is in the early seedling and germination stages, it is convenient to understand the effect of salinity, as these stages are more prone to damage (Munns and Gilliham, 2015). Salt stress damages plant growth and developmental stages through the introduction of different other stresses like water stress, nutritional imbalance, and cytotoxicity caused by increased uptake of sodium (Na^+^) and chloride ions (Cl^−^). In addition, salt stress is normally followed by oxidative stress due to the formation of reactive oxygen species (ROS) (Tsugane et al., 1999; Hernández et al., 2001; Ісаєнков, 2012).

There are two major phases in which plant’s responses to salt stress have been divided. In the first phase, within minutes to days, an ion independent decline in growth takes place which leads to closure of stomata and hampering of cell expansion majorly in the shoot area (Munns and Passioura, 1984). After few days or even weeks, a second phase occurs and causes an increase in the levels of cytotoxic ions. It then leads to slowing of metabolic processes, premature senescence, and eventually cell death (Munns, 2008; Roy, 2014).

**Figure 2:**
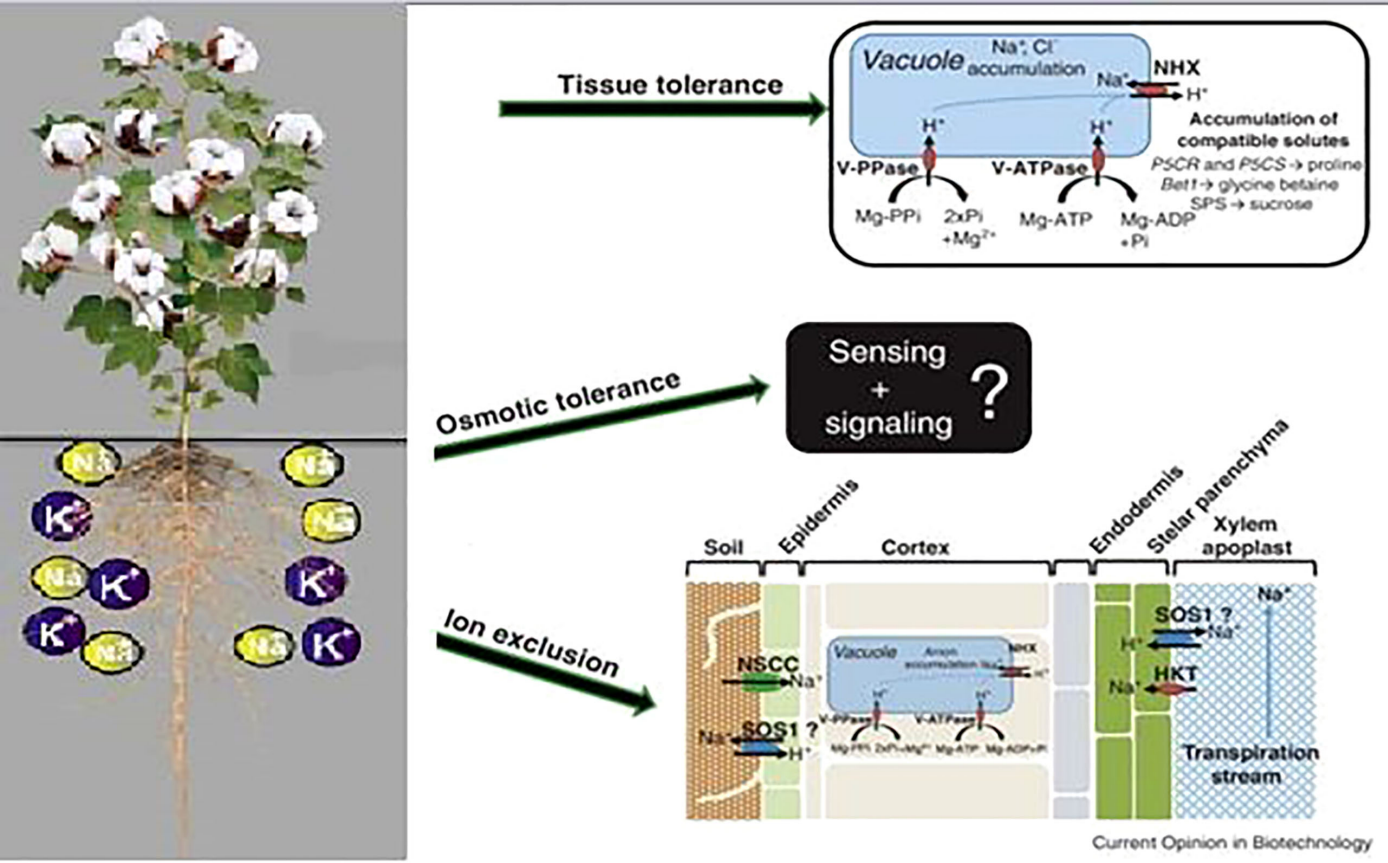
Mechanisms of tissue tolerance, osmotic tolerance and ion exclusion in a plant (Roy, 2014).

Tolerance to abiotic stress is controlled by the assembly of different molecular and physiological mechanisms like osmotic tolerance, ionic tolerance, and tissue tolerance (Rajendran et al., 2009). The plant exhibits osmotic tolerance comparatively quickly in which a prompt decrease in stomatal conductance to conserve water is noticed. It utilizes quick long distance signaling mechanisms (root to shoot) (Ismail et al., 2007), (Zimmermann et al., 2009), which do not differentiate between osmotic effects generated by NaCl, mannitol, KCl, or polyethylene glycol (Yeo et al., 1991; Chazen et al., 1995). When salt enters into the root system, many signal cascades get activated that help in developing ionic tolerance by limiting the net influx of Na^+^ into the roots and reducing the net Na^+^ translocation. Finally, toxic ions are compartmented into vacuoles to evade their injurious effects on cytoplasmic processes. This mechanism is called tissue tolerance. These different mechanisms have been observed and studied in many plants and they are further explained in **Figure 2**.

However, there are some differences between halophytes and glycophytes in terms of their tolerance. These are primarily due to the greater strength and vigor of the working mechanisms in halophytes as compared to the glycophytes (Flowers and Colmer, 2008; Maathuis et al., 2014). Salinity creates a plight for plants, as it leads towards water and osmotic stress due to higher levels of inorganic minerals in the environment. Regardless of years and years of research, one of the most inexplicable questions related to salinity stress in plants remains the mechanism of the entry of Na^+^ and Cl^−^ into the roots.

Cotton is relatively more tolerant to abiotic stresses; however, cotton is susceptible to salt stress at critical stages like germination, flowering, boll formation, resulting in negative effects on the biomass production and substantial drop in fiber produce by nearly 60% (Ahmad et al., 2002; RK, 2009).

## 4. Morphological effects on cotton plant in response to salt stress

Generally, salinity stress seriously limits cotton growth and development, for example, reduction in plant stature, fresh and dry weights of roots and shoots, number of nodes, leaf area index, stomatal conductance, transpiration rate, photosynthesis, yield, canopy and root development, and fiber quality (Loka et al., 2011).

### 4.1. Root system

Some plants have the potential to accelerate root growth in response to abiotic stresses at an early stage, resulting in longer root systems that can collect water from deeper soil. Abiotic stress tolerance in crops has been linked to increased root growth calculated as length, volume, weight, and density of plant roots. Some crops like cotton can have tap roots with length 10 times the height of the above-ground plant (Larcher, 2003). The ionic influx in the roots and its translocation to the shoot is essential for plant growth. Comparatively less suppression of root growth than shoot growth may be credited to lower retention of sodium ions in roots. This could also be due to the expression of some genes under salt stress. Studies have shown that some salt associated genes are up-regulated under salt stress for 3 hours or 6 hours like *CP_1_*, *MPK_3_*, *SOS_3_*, *AB_11_*, *CIPK_6_*, and *STZ/ZAT_10_* (Yao et al., 2011). Root growth was reduced significantly in different types of soils as salinity increased, however, the suppression of root growth, and fresh and dry weight was highest in clay, loam, and intermediate soils, and lowest in sandy soil (Soliman et al., 1980).

### 4.2. Germination

In cotton, some stages are more susceptible to salt stress as compared to others, like, germination, emergence, and young seedling stage (Ahmad et al., 2002). Cotton germination and emergence stages are also delayed in response to saline stress (Khorsandi and Anagholi, 2009; Ma et al., 2011). In some studies, cotton plant germination was delayed by up to 4–5 days when exposed to salinity stress of 15–20 dS m^-1^, as compared to normal plants. Poor germination results in decline of plant population, which eventually leads to a significant decline in cotton yield (Saqib et al., 2002). Seed germination recorded in sand culture was 68-89% at 150 mM and 24-40% at 250 mM salt as compared to soil where it was 72-89% at 15 dS m^-1^ and 20-53% at 25 dS m^-1^ (Khan et al., 1995). As the salt concentration was increased, germination percentage showed decline which ranged from 17-100% depending upon the concentration of salt (Sattar et al., 2010). However, cotton germination was not affected under low concentration (≤1.2%) of salt but higher concentrations (≥1.2%) affected the germination adversely (Wang et al., 2007). Both delay and reduction in germination was observed in cotton grown in nutrient medium, under salt concentration of 200 mM (Kent and Läuchli, 1985).

### 4.3. Shoot system

Cotton growth and development is seriously affected by salinity level above than 7.7 dS m^-1^. Even though cotton is categorized as a comparatively more salt-tolerant crop than other major crops, still its vegetative growth is affected negatively at higher concentrations of salt. Salinity stress leads a reduction in shoot/root ratio as shoot growth is more sensitive to salt stress than root growth (Khan et al., 1995). Salinity manifests itself in a variety of ways. Normally cotton plants under salinity stress appeared to be similar to plants under drought stress, showing symptoms like smaller and dark blue/green leaves. This happens due to increased osmotic pressure in the soil solution, which causes physiological dryness and the accumulation of one or more elements that may obstruct nutrient and water uptake (Praharaj and Rajendran, 2004). Leaves become hard, brittle, and necrotic, resulting in underdeveloped growth and yield reduction, as salinity level increases. Other problems linked with salinity are high osmotic pressure, less availability of nitrogen, phosphorus, manganese, copper, zinc and iron, low microbial activity, and poor air and water movement (Pessarakli, 2001). Six-leaf stage is proved to be most sensitive to stress as compared to other stages (Khan et al., 1998).

### 4.4. Yield and yield related components

Many field studies have been carried out to look into the effects of drought or salt on lint yield, the most significant end product of cotton production. Physiological and/or morphological characters have been reported to show negative correlations with yield. As salinity level increases, a decline in cotton yield has been observed because of the reduced number of bolls and low boll weight. Reduction in the number of mature bolls is due to several factors such as delayed flowering, low fruit-bearing positions, and relative increase in flowers and bolls shedding under salinity conditions (Anagholi et al., 2005). Up to 60-87% synthesized sucrose is translocated from Subtending Leaf of Cotton Boll (LSCB) to developing bolls in cotton and it has a very significant role in cotton yield. Though sucrose accumulation in LSCB remains unchanged, its effective translocation towards developing bolls is delayed under salt stress that eventually leads to reduced boll weight (Peng et al., 2016). Almost 50% reduction in cotton yield was observed under 17.0 dS m^-1^ salt concentration (Maas and Hoffman, 1977), yet, moderate level of salinity showed to cause no harmful effects on growth. As the level of salinity was increased, shedding and premature leaf senescence occurred (Rathert, 1983). Some other studies (Ashraf and Ahmad, 2000; Ashraf, 2002) also reported almost 50% reduction in yield when salinity was increased from 7.7 dSm^-1^ to 17.0 dSm^-1^.

### 4.5. Fiber quality

Although fiber quality is a genetic trait, it is affected by the environment too. Studies have shown that salt stress reduced the fiber strength, length, and maturity but increased the fiber fineness. Micronaire values also showed decreasing trend when sodium ions percentage was increased (Longenecker, 1974). Increase in electrical conductivity (EC) has an impact on cellulose deposition, photosynthesis process, and sugar transport which indirectly affects fiber maturity. Due to less cellulose deposition cross-sectional area is decreased inevitably and production of mature fiber is reduced (Yfoulis and Fasoulas, 1973). Nevertheless, a study showed that under salt level of 0.42%, an increase in fiber length and decrease in fiber fineness and elongation was observed (Ye et al., 1997). One of the most significant factors affecting fiber quality is the timing of the stress. Two American cotton cultivars of Acala 4–42 and Deltapine Smooth leaf were introduced with early stress during flowering season and then again soon after flowering. No effect on quality of fiber was observed due to early stress but the stress given after flowering stage reduced the fiber quality (Marani and Amirav, 1971).

### 4.6. Seed oil content

Cottonseed normally contains approximately 20-23% of oil (Dowd et al., 2010). Another studies showed that dried cottonseed comprises of 28-44% of oil content having both saturated and unsaturated fatty acids (Shang et al., 2016). It is usually presumed that increased salt stress results in the reduction of seed oil content (Khan and Gregori, 2002). It was observed in six genetically dissimilar cotton lines having different levels of tolerance to salt that increase in the salt level of the growth medium led to decrease in oil content (Ashraf and Ahmad, 2000). In another study, results showed decrease in oleic and linoleic acid content of cottonseed obtained from salt stress exposed plants (Ahmad et al., 2007). However, genotypes with higher tolerance to salt yielded a higher percentage of seed oil content as compared to salt susceptible genotypes. In case of cotton, levels of salt tolerance vary within and between different cotton species as there are variety of plant characteristics associated with each species. Such diversity of genomes would be of substantial practical value in improving salinity tolerance of cotton through selection and breeding as maximum proportion of this diversity is genetically additive and based in nature.

## 5 Physiological mechanisms of cotton in response to salt stress

### 5.1 Photosynthesis

Photosynthesis, which is controlled by stomata for CO_2_/water exchange and photosynthetic activity in mesophyll cells, determines plant productivity. The rate of photosynthesis is lowered at high salinity levels due to disruption in photosynthetic activities and apparatus. Osmotic stress was reported to be caused by increased NaCl levels in the soil, which limited cell expansion and reduced stomatal aperture size, limiting photosynthetic activity in cotton. The first observable effect of salt stress is the reduction in leaf surface area. At initial stages, reduced leaf surface area due to limited cell extensibility is more significant than lower photosynthetic rate per unit area (Shabala and Lew, 2002). Reduced photosynthetic activity is also due to lower Ribulose bisphosphate (RuBP) carboxylase efficiency under restricted RuBP supply, PSII sensitivity, and less regeneration capacity of RuBP (Seemann and Sharkey, 1986). Salt stress is known to interfere with photosynthetic biochemistry by disorienting the chloroplast lamellar system and causing chloroplast integrity loss, resulting in decreased photosystem activity in cotton plants. Salt stress may have a secondary effect on photosynthetic enzymes, mediated by lower CO_2_ pressure in the leaves due to stomatal closure.

According to a recent study, the decline in photosynthesis is related to decrease in chlorophyll contents and variation in chlorophyll ultrastructure (Meng et al., 2011). Cotton cultivars showed a significant drop in chlorophyll contents (a and b) when salt level was increased (Zhang et al., 2014b). This decline could be related to the suppression of certain enzymes involved in chlorophyll synthesis (Lee et al., 2013). As a result, among physiological parameters, chlorophyll contents could be considered as an excellent indicator for selecting salt-tolerant cultivars. Plants subjected to salinity stress showed a decrease in the expression of carotenoids biosynthesis genes that are strongly linked to a reduction in photosynthetic rate, which eventually influenced the yield negatively (Shah et al., 2017). Previous researches also reported a considerable decrease in carotenoids content in cotton genotypes as salt stress increases (Zhang et al., 2014b).

### 5.2. Concentration of inorganic ions

Soil salinity inhibits plant growth primarily through three mechanisms: osmotically induced water stress, specific ion toxicity caused by high levels of sodium and chloride ions, and nutrient ion imbalance caused by high levels of Na^+^ and Cl^-^, which reduces the uptake of K^+^, NO-, PO_4_^3-^ etc (Greenway and Munns, 1980). Various studies have reported changes in inorganic ion concentrations when plants are exposed to salt stress. Different responses have been recorded, ranging from an immense rise in Na^+^ and Cl^-^ ions concentrations to a reduction in Mg^2+^, K^+^, and Ca^2+^ ions (Jafri and Ahmad, 1995).

Excessive Na+ ion accumulation has lethal effects on plant physiological processes and also causes reduction in water availability. A considerable increase in the concentration of Na^+^ and Cl^-^ ions was reported with decreased K^+^/Na^+^ ratio in cotton leaves. Salt tolerant plants controlled the exclusion of Na^+^ ions through their roots, whereas plants that were unable to maintain Na^+^ homeostasis were classified as sensitive (Pervaiz et al., 2007). According to several studies, rather than Na+ exclusion, upkeep of optimum ratio of K+/Na+ ions defines the plant performance under salt stress (Ding et al., 2010; Dai et al., 2014). Besides sodium and chloride ions, N, Zn and Mn were also increased under salinity while Ca^2+^, P, and S levels stayed consistent. However, concentrations of K^+^, Mg^2+^, Fe, and Cu ions were decreased significantly under salt stress (Higbie et al., 2010).

The chloride ion is more dangerous in a variety of species as compared to sodium ion including cotton (Luo et al., 2002; Tavakkoli et al., 2010). With higher concentrations of NaCl in the soil environment, the concentrations of Na^+^ and Cl^-^ in cotton roots, xylem sap, and leaf also increase (Hirayama and Mihara, 1987). When Na^+^ and Cl^-^ ions move into cells, they disrupt the ion balance in the cytoplasm, especially the Ca^2+^ balance. When Na^+^/Ca^2+^ ratio increases, a considerable concentration of Na^+^ and Cl^-^ can replace bound Ca^2+^ in the cell membrane system that can lead to impairment of structural integrity and function of membrane. Finally, a sudden increase in free Ca^2+^ in cytoplasm can lead to the weakening of cellular metabolism (Hirayama and Mihara, 1987). However, there are different mechanisms by which plants can adapt to salinity such as Na^+^ or Cl^-^ exclusion, osmotic stress tolerance, and tissue tolerance to accumulated Na^+^ or Cl^-^ (Munns and Tester, 2008).

Ionic imbalance limits the access and transport of nutrients within plants because of Na^+^ and Cl^-^ ions competition with other nutrients like Ca^2+^, NO_3_^-^, and K^+^ and consequently reduces the concentration of Ca^2+^, Mg^+2^, K^+^, N, and P in leaves and roots (Mansour et al., 2005). Though, several studies have found that the concentrations of S, Ca^2+^, and K^+^ in leaves remained stable, resulting in lower K^+^/Ca^2+^ or K^+^/Na^+^ ratios (Abd Ella and Shalaby, 1993).

## 6. Mechanism of salinity tolerance in cotton

In order to continue their growth and development in highly saline soils, plants develop different physiological and biochemical mechanisms which majorly are; (i) ion homeostasis and compartmentalization, (ii) ion transport and uptake, (iii) biosynthesis of osmo-protectants and compatible solutes, (iv) activation of antioxidant enzyme and synthesis of antioxidant compounds, (v) synthesis of polyamines, (vi) generation of nitric oxide (NO), and (vii) hormone modulation. Some of these are discussed below.

### 6.1. Ion transport

In order to adapt to a saline environment, halophytes are known to accumulate high quantities of ions (Na^+^ and Cl^-^) in their tissues (Flowers et al., 1977), whereas mesophytes are usually identified as the ones which limit the uptake of these ions (Greenway and Munns, 1980; Wyn Jones, 1981). Salt tolerance in crop species and specific injury from ions is accounted by the preferential accumulation of either Na^+^, Cl^-^, or both ions, rather than osmotic stress, which was thought to be the key factor for salt sensitivity (Grattan, 1996; Jacoby, 1999). The ratio of K^+^/Na^+^ has been proved to be an effective mean for selection for salt tolerance in various crops, out of many different suggested physiological traits (Giriraj et al., 1976; Janardhan et al., 1976; Schleiff, 1978; Murthy, 1979). Shoots of cotton plats exposed to saline conditions showed low accumulation of Na^+^ and high K^+^ (Eaton and Bernardin, 1964). Salt tolerant plants decrease the influx of Na^+^ from roots, compartmentalize the exciting Na^+^ present in the cytosol to vacuole, and exit of Na^+^ from root cells to maintain the Na^+^/K^+^ ratio (Keisham et al., 2018). Plant cells use primary active transport facility through Na^+^/H^+^ antiporters i.e., Na^+^/H^+^ exchangers (NHX) for vacuolar compartmentalization, H^+^-ATPases, channels i.e. K^+^ channel (AKT1), Salt overly sensitive (SOS) pathway for Na^+^ exit and for keeping high K^+^/Na^+^ ratio in the cytosol, and co-transporters mediated secondary transport i.e., High affinity Na^+^ transporter (HKTs) and High affinity K^+^ transporter (HAK5) (Conde et al., 2011; Zhao et al., 2013). The key selection criteria for selection for salt tolerance is maintaining high ratios of Ca^2+^/Na^+^ and K^+^/Na^+^ in response to salinity stress. Up and down regulation of some genes was shown in salt tolerant genotypes of cotton such as; up-regulation of *GhHKT1* and *GhNXH1* and down regulation of *GhSOS1*, *AKT1* and *HAK-5* maintained the high selective absorption of K^+^ and Na^+^. This proves that salt tolerance in cotton is greatly associated with regulation of K^+^ and Na^+^ ions by Na^+^ ion compartmentalization into vacuole as compared to uptake of K^+^ ion (Wang et al., 2017b). Salt

**Figure 3:**
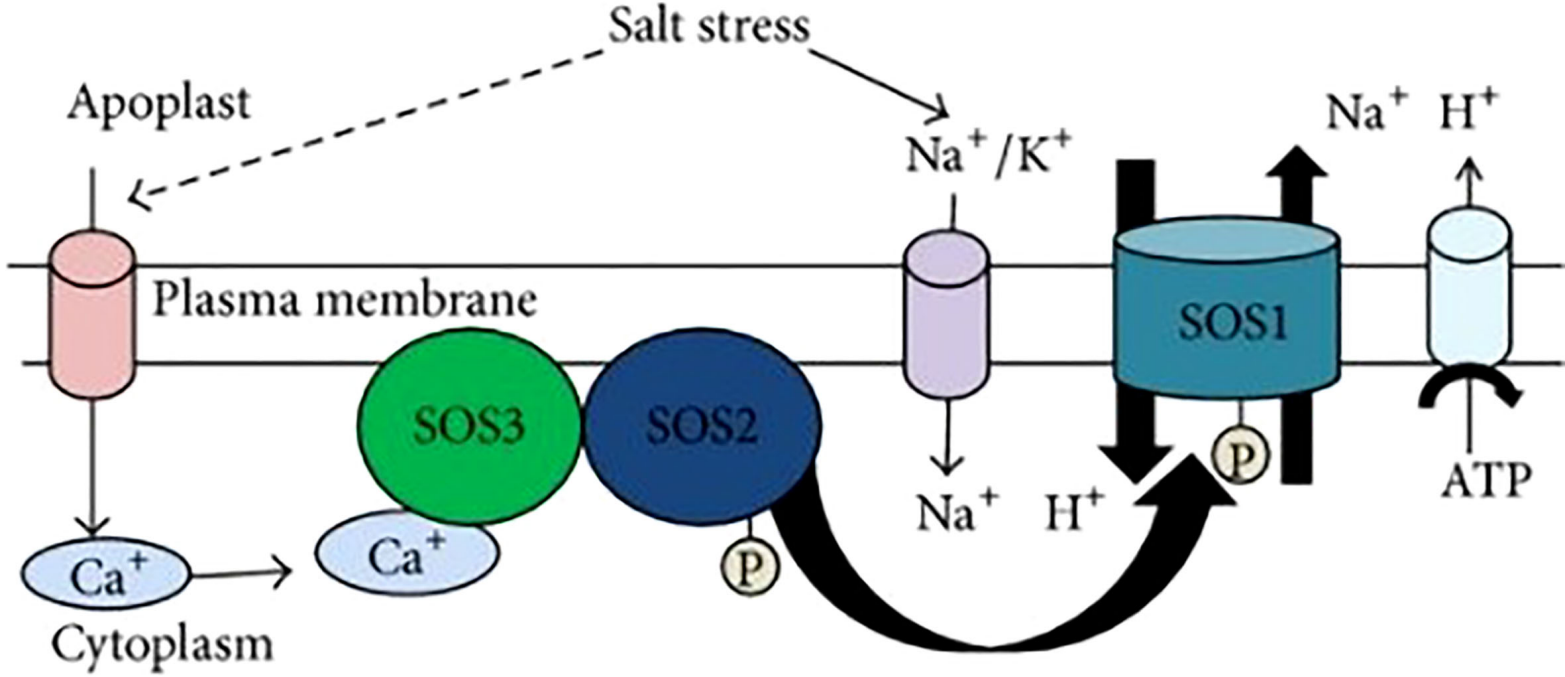
SOS Pathway (Gupta and Huang, 2014).

Overly Sensitive (SOS) pathway also contributes towards ion homeostasis and salt tolerance. It contains three major proteins (**Figure 3**), SOS1, SOS2, and SOS3.

### 6.2. Organic solutes

Accumulation of osmo-protectants such as polyamines, sugars, glycine betaine, amino acids, and polyols for metabolic adjustment greatly contributes to salt tolerance in order to overcome salt stress. Organic ions are compartmentalized within the vacuole to balance the osmotic potential within it. These organic solutes help to maintain cell turgor, create a gradient force for water uptake, and stabilize the membranes and proteins against the denaturing activity of detrimental solutes and salts (Naidoo and Naidoo, 2001; Rontein et al., 2002).

Glycine betaine is an essential organic solute that functions as an osmoprotectant and accumulates under conditions such as drought/water stress, salt stress, and temperature extremes (Quan et al., 2004). Contribution of glycine betain towards osmotic adjustments under saline conditions has been reported by many researchers (Khan et al., 1998). A high level of glycine betaine was observed as an indicator of salt tolerance in transgenic lines of cotton in which an enzyme Choline Monooxygenase (CAM) was genetically engineered. Choline Monooxygenase is responsible for the conversion of choline into betain aldehyde during the catalytic pathway. Furthermore, the betain aldehyde is catalyzed by various enzymes into glycine betain (Zhang et al., 2009).

Proline, an amino acid, plays a very vital role in stabilizing the RUBISCO enzyme and stimulates its functionality even in the presence of NaCl. After being treated with sea water, the levels of free proline in *G. hirsutum* and *G. arboreum* increased (Ahmad and Abdullah, 1982). A study found a 36% rise in proline content in the roots of treated plants compared to control and a 121% increase in proline level in the leaves after treatment with NaCl (Meloni et al., 2001). In contrast, another study stated inconsistent changes in proline or hydroxyproline content in cotton in response to salinity stress. Thus, proline concentration is insufficient to meet the osmotic adjustment; yet, if restricted to the cytosol, it could play an important role (Golan-Goldhirsh et al., 1990).

### 6.3. Antioxidants

Increased activity of antioxidants is generally supposed to be an indicator of salt tolerance in plants (Noreen and Ashraf, 2009). The generation of reactive oxygen species (ROS) such as superoxide, hydroxyl radical, and hydrogen peroxide is stimulated by salt stress. The ROS are neutralized by intracellular antioxidants under normal conditions, but during salt stress, excessive accumulation of ROS incites oxidative stress and brutally disrupts normal metabolism, causing protein breakdown and nucleic acid mutation (Czégény et al., 2014). Plants have two types of antioxidant systems to combat salinity-induced oxidative damage: enzymatic and non-enzymatic. The enzymatic oxidant system comprises superoxide dismutase (SOD), peroxidases (POD), glutathione peroxidase, and catalase (CAT) accompanied by enzymes of ascorbate-glutathione peroxidase [glutathione reductase (GR) and ascorbate peroxidase (APX)]. Due to its function in the regulation of O_2_^-^ and H_2_O_2_ concentrations, SOD is considered a significant antioxidant enzyme. APX and CAT has a proficient ability of H_2_O_2_ scavenging. During the scavenging process, APX and CAT play an essential role in the presence of SOD as CAT dismutates H_2_O_2_ to H_2_O, while APX does the same through the ascorbate-glutathione pathway (Zhang et al., 2014b).

The oxidation of numerous biological substances such as proteins, lipids, DNA, and RNA occurs when ROS such as hydroxyl radical (HO•) and singlet oxygen accumulate in the cell (Tripathy and Oelmüller, 2012). Plants have evolved antioxidative mechanisms to deal with the formation and accumulation of reactive oxygen species (ROS). These mechanisms include: (i) non-enzymatic antioxidants, like ascorbic acid, carotenoids, a-tocopherol, reduced glutathione, and flavonoids; and (ii) enzymatic components, like ascorbate peroxidase (APX), catalase (CAT), guaiacol peroxidase (GPX), glutathione reductase (GR), monodehydroascorbate reductase (MDAR), and dehydroascorbate reductase (DHA). Despite the fact that antioxidative mechanisms are the plant’s first choice for dealing with production of ROS, a recent study reported that the balance between ROS production and antioxidative enzyme activity under drought stress in cotton controls whether oxidative signaling and/or injury occurs (Zhang et al., 2014c).

Two cultivars of cotton (‘84-S’ and ‘M-503’) having different levels of tolerance to salt stress were assessed for biochemical and physiological responses against combination of different abiotic stresses including salt stress. When compared to control conditions, the sensitive cultivar 84-S displayed lower CAT and peroxidase (POX) activities under stress conditions causing higher H_2_O_2_ accumulation and oxidative stress induced lipid peroxidation. On the other hand, under stress conditions, the tolerant cultivar M-503 sustained constitutive levels of superoxide dismutase (SOD) and APX enzyme activities while increasing CAT, POX, and proline content levels (Sekmen et al., 2014). Another study stated that under PEG 6000, supplemental Zn improved photosynthetic rate, chlorophyll a and b content, and biomass of cotton significantly increased (Wu et al., 2015).

## 7. Screening or selection for salt tolerance

Methods for screening soil salinity are quite complex. Because fields with a uniform salinity are not frequently available, evaluating for salinity tolerance in the field is difficult and often inaccurate. The substantial experimental errors caused by variations in the levels of soil salinity across a field lead to minimal progress in salt tolerance enhancement (Abdelraheem et al., 2015a). Nonetheless, if a field with homogeneous soil salinity can be located, it can be very useful in screening a large number of cotton germplasm and selecting for their relative salt tolerance. For that reason, screening of germplasm by using pot culture is a suitable method, as it allows for the creation of graded salt levels by irrigating the plants with known salinity solutions (Abdelraheem et al., 2016a). But still due to high level of variation in physical and chemical properties of soil, it is hard to tell if the plant is growing at the required level of salinity (Abdelraheem et al., 2016b). The most sought after solution for that problem is screening of germplasm for salinity tolerance in hydroponics (water culture). Plants are grown in hydroponics with a known amount of salinity, allowing researchers to assess the absolute effect of salt on plant development and the threshold concentration needed to cause a reduction in plant growth (Abdelraheem et al., 2015b).

Using a hydroponic setup, a study was performed to assess multiple genetic mapping populations for drought and salt tolerance, including a multi-parent advanced generation inter-cross (MAGIC) population of 550 lines and an association mapping panel of 376 Upland accessions of cotton. The seedlings were transferred from pots to a hydroponic system where they were treated with salt or drought treatments using PEG, NaCl, or water. Then, fresh and dry weights of the shoots and roots as well as plant height were measured. Shifting from pots to hydroponic system allowed improved and extensive study on growth and architecture of roots, which has been neglected in the past, as it was difficult to extract and evaluate roots by using old screening methods. Also hydroponic system allowed examination of the responses in older plants which could not be possible in small pot system as seedlings can only be treated for a little period of time like 3-4 weeks there (Abdelraheem et al., 2018).

Identifying and evaluating salt-tolerant plants based on plant biomass or yield, whether in the laboratory or in the field, is a time-consuming and hectic task. Hence, a number of physiological indicators have been suggested for identification and assessment of salt tolerance. For selection of salt tolerance in cotton, the K^+^/Na^+^ ratio has been utilized as an effective criteria (Akhtar et al., 2010). The best indicators of salinity for cotton cultivar selection were reported to be seed germination, decrease in leaf area, Na^+^ and K^+^ content, and seedling growth, however chlorophyll and proline contents were not effective for accurate assessment of salinity tolerance (Munis et al., 2010). By using a transducer, emergence force applied by germinating seedlings of cotton was measured. Increasing the level of salinity decreased the emergence force and lengthened the time needed to develop the maximum force (Sexton and Gerard, 1982). Tests like these could be utilized to measure the salt tolerance of germinating seedlings (Stewart et al., 2009). Pollen grain germination has also been used for salt tolerance screening (Fafu et al., 1997).

Zhang et al. used 88 simple sequence repeat (SSR) markers to study 47 upland cotton accessions, including 23 salinity-tolerant and 24 salinity-sensitive cotton materials. They discovered 338 alleles at 88 SSR loci, with an average of 3.841 alleles per locus. Out of these alleles, 333 alleles were identified in salinity tolerant germplasm and 312 alleles in salinity sensitive germplasm. In the salinity tolerant germplasm, average effective number of alleles (Ne), mean polymorphism information content (PIC), and average genotype diversity index (H′) were 2.929, 0.613, and 1.083, respectively, while in the salinity sensitive germplasm, they were 2.883, 0.605, and 1.071. Salinity tolerant and salinity sensitive germplasms had comparable similarity coefficients. In salinity tolerant germplasm, they ranged from 0.530 to 0.979, with a broader range than in salinity tolerant germplasm. The varieties were divided into three categories: one large group and two smaller groupings. The significant genetic similarity coefficients found in Chinese salinity-tolerant germplasm suggested that the group had narrow pedigrees (Zhang et al., 2010). As a result of the limited germplasm resources available and the complexity of tolerance mechanisms in cotton, little progress has been made in developing significantly salt-tolerant cultivars using traditional methods. However, such research is also vital because it not only enhances our knowledge of salt-tolerant cotton resources, but it is also the most direct and reliable technique of locating salt-tolerant cotton germplasm.

## 8. Sources of salinity tolerance in cotton

Out of four cultivated species of cotton, *G. barbadense* has some desired traits like higher levels of tolerance to different types of biotic and abiotic stress (Zhang et al., 2014a). But *G. barbadense* is cultivated on very limited areas and due to hybrid breakdown, stress tolerance have not been transferred into commercial American cotton successfully (Zhang et al., 2014a). Furthermore, due to extensive artificial selection for economically significant traits during the domestication and breeding process, genetic diversity of Upland/American cotton is quite low (Wendel et al., 1992; Abdalla et al., 2001). To screen germplasm of cotton for salt and drought tolerance, a number of research studies were performed in the greenhouse. Up to 367 cotton accessions were screened for salinity and drought tolerance in greenhouse. As a result, 45 accessions were reported to be salt tolerant while 24 were reported to be drought tolerant. Moreover, there were some accessions that showed tolerance to both stresses (Abdelraheem et al., 2016a). Another study screened more than 1500 recombinant inbred lines (RILs) from diverse genetic mapping populations and identified that tolerance to both drought and salinity stress was in intraspecific and interspecific cotton (Abdelraheem et al., 2015a; Abdelraheem et al., 2015b; Abdelraheem et al., 2016b; Abdelraheem and Zhang, 2016). Barrack et al. assessed four introgressed Upland cotton lines from a *G. hirsutum* × *G. barbadense* backcross inbred line population that were sown in two different soil types, i.e., an organic soil and a loam soil with 200mM NaCl for a time period of three weeks. Data of various traits was collected such as, leaf size, chlorophyll contents and fluorescence, main stem node number, plant height, shoot biomass, internode length, and number of fruiting sites. Results showed genotypic variation, that the optimum time for screening of cotton for salinity tolerance is seedling (Barrick et al., 2015). Another study validated that *G. barbadense* is more tolerant as compared to *G. hirsutum*, when theirs seeds are germinated under 200 mM NaCl salinity level (Tiwari et al., 2013). Furthermore, Niu et al. conducted an experiment on five genotypes of cotton, two Pima cotton (‘Pima Cobalt’ and ‘Pima S-7’) and three Upland cotton (‘DN 1’, ‘DP 491’, and ‘FM 989’), using concentrations of 100 and 150 mM NaCl and 70 and 111 mM Na_2_SO_4_. Compared to control conditions, all genotypes showed a considerable reduction in growth, but there was no major difference between Pima and Upland cotton identified in their response to salinity stress (Niu et al., 2013).

## 9. Breeding for salt tolerance *via* marker-assisted selection (MAS)

Numerous researches have been undertaken since the advent of genetic markers and marker-assisted selection (MAS) technology to find genes or quantitative trait loci (QTLs) affecting salt tolerance in various plant species during their different developmental stages. The goal of these investigations was to see if marker-linked QTLs or genes could be used in MAS breeding for salt tolerance. However, by using MAS technology, only limited progress has been made in developing salt-tolerant cultivars. This is in contrast to the widespread use of MAS in crop breeding for a variety of simple characters in many different crop species. Nevertheless, by using molecular markers, QTLs responsible for increased cotton produce under abiotic stress conditions have been identified. These QTL will aid in the understanding of the genetic basis for drought and salt tolerance, as well as the breeding of drought and salt-tolerant cultivars using MAS.

### 9.1. QTLs associated with salt tolerance

Since 1994, when the first linkage map in cotton was constructed (Reinisch et al., 1994) using 705 loci of restricted fragment polymorphic markers (RFLPs) from an interspecific cross between Upland and Pima cotton, many linkage maps have been constructed to dissect various complex traits in cotton using different types of markers. However, most of these linkage maps have been used to discover QTL for yield and yield components, as well as fiber quality under normal production conditions, with only a few studies identifying QTL for abiotic stress tolerant traits.

Several researches have been conducted in numerous crop species over the last two decades to find QTLs that affect the diverse plant responses to salt stress at various developmental stages, including reproduction, seed germination, and seedling and vegetative growth. Oluoch et al. examined salt tolerance in a hydroponic environment for two weeks at 150 mM of NaCl, using a population of 188 F_2:3_ progeny obtained from a cross between Upland and an accession of a wild type belonging to *G. tomentosum* Nutt. ex Seem. In at least two environments, 11 consistent QTLs were identified on eight chromosomes (9, 11, 15, 16, 21, 23, 24 and 26). One of the major QTL reported was Qrl-Chr16-1 elucidating the phenotypic variance of 11.97 and 18.44% in two environments. Results showed that in AD genome of allotetraploid cotton, genes contributing towards salt tolerance were mostly derived from D subgenome as 10 out of 11 QTLs detected were located on the D subgenome (Oluoch et al., 2016). Another study was conducted to locate salt and drought tolerance QTLs in an introgressed Upland cotton population that was developed under controlled field and greenhouse conditions, by utilizing 1004 polymorphic DNA marker loci comprising 481 single nucleotide polymorphism (SNPs) and 523 SSRs. A total of 165 QTLs were reported to be found across most of the chromosomes, with each QTL elucidating 5.98-21.43% of phenotypic variation. Furthermore, 15 QTLs that were common to salt and drought stress tolerances were identified on 12 chromosomes. Chromosome c5 had a QTL cluster for plant height which was identified in both greenhouse and field conditions under drought stress but same cluster was observed in greenhouse only under salinity stress (Abdelraheem et al., 2019).

More QTL mapping for tolerance to abiotic stress using various techniques is required because of restricted number of QTL for drought and salt tolerance in cotton. Furthermore, surplus markers and candidate genes are needed for tolerance to abiotic stresses besides these progresses (Tiwari et al., 2013; Rodriguez-Uribe et al., 2014; Abdelraheem et al., 2015b).

Genotyping-by-sequencing (GBS) has been employed in a variety of crops, including cotton, as a less expensive option for detecting thousands of genome-wide SNP markers across many individuals from various populations (Elshire et al., 2011; Poland and Rife, 2012; Gore et al., 2014; Abdelraheem and Zhang, 2016). Moreover, measuring genetic variations of SNPs between different populations can also be done by utilizing and developing SNP chips for identification of high levels of polymorphisms. Permanent mapping populations for salinity tolerance in cotton should be developed and used in QTL mapping because replicated experiments utilizing same genetic populations are imperative to identify genetic variation consistently and to comprehend the genetic basis for abiotic stress tolerance based on QTL mapping. A study directed that improvement can be made in this field using a large RIL population to measure the genetic relationship of abiotic stress tolerance (Asins, 2002).

Association analysis was used in cotton genotypes to find out eight SSRs linked to salt tolerance, out of which, two were highly linked and explained phenotypic variations from 7.82 to 6.26% (Zhao et al., 2016).

Proteomic approaches can be used to detect proteins that are associated with salinity tolerance. For identification of 58 differently abundant salt responsive proteins in cotton seedling leaves, iTRAQ was used in a study. Phospho ethanol amine, phosphate-related differentially abundant proteins (DAPs), 14-3-3-like protein E and N-methyltransferase 1 were induced in salinity stress. Twenty-nine salt responsive proteins were identified to be genotype-specific, with 62.1% and 27.6% of them being linked to chloroplast and defense response, respectively (Gong et al., 2017). These outcomes put forward effectiveness of MAS in identification and development of genotypes with salinity tolerance.

Using a set of 109 accessions of cotton, marker trait associations were evaluated. Screening was done with the help of 250 SSR markers for polymorphism. Results revealed that markers BNL3140, BNL3103 and NAU478 were definite to be linked with salt treatment (Saeed et al., 2014). In 108 elite cotton lines, twenty-six markers on 14 chromosomes and 177 SSR markers were found to be linked to *Verticillium* wilt resistance (Baytar et al., 2017). However, due to the low marker densities and populations employed in earlier association studies, QTL mapping for stress tolerance has remained inadequate.

In cotton, whole genome exposure has been accomplished by genome wide association study (GWAS) by the use of economical genome-wide DNA markers (Zhu et al., 2008). This method has a stronger statistical power than biparental mapping for detecting significant QTL with high resolution to explicate more phenotypic variation. On salinity tolerance, very few studies have been performed due to its complicated phenotyping in cotton. Even though these studies had greater number of genotypes, still most of them utilized low genome coverage of SSR markers. Recent studies assessed 323 Upland cotton accessions for abiotic stress tolerance and carried out GWAS using 106 SSR markers. Salinity tolerance was reported to be linked with three SSR markers (Jia et al., 2014). Another GWAS was performed in 304 Upland cotton genotypes for salinity tolerance using 145 SSR markers. Results reported approximately 95 loci to be significantly linked with traits contributing towards salt tolerance (Du et al., 2016). Lately, nine candidate intron length polymorphisms (ILPs) markers were confirmed in upland cotton for salinity by using association mapping under controlled conditions (Cai et al., 2017). A total of 74 SSR markers were utilized and 148 loci were found, out of which eight SSR sites were found to be linked with salinity tolerance with the help of association analysis (Zhao et al., 2016).

#### 9.1.1 Functional genomics tools for improvement of salinity tolerance in cotton

Sequencing of genomes of tetraploid cotton (Zhang et al., 2015), its sub-genomes as well as other *Gossypium* species, such as, *G. ramondii* (Wang et al., 2012), *G. barbadense* (Liu et al., 2015b), and *G. arboreum* (Li et al., 2014), and lately genome wide resequencing of 352 cotton genotypes (Wang et al., 2017a), 243 diploid genotypes (Du et al., 2018), and 419 accessions (Ma et al., 2018), provides a wide-ranging genome wide evaluation to isolate genes, genomic regions, and SNPs, which can eventually be used for salt stress tolerance in cotton. Cotton genome sequencing and resequencing provide a foundation for identifying genes and genome architecture in cotton. Cotton functional genomics advances can aid in the investigation of biologically active regions and genes across the entire genome. We can now investigate salt stress tolerance in cotton using SNP array platforms, fine and high-density genomic mapping, transcript abundance, and epigenetic changes. Successively, sub-genome species sequencing and the construction of dense and ultra-precise genetic linkage maps will offer a platform for gene isolation, gene mapping, and high-throughput development of markers for stress tolerance (Ashraf et al., 2018).

With the advancements in next generation sequencing (NGS) techniques and in silico methods, whole genome (2.5 Gb) SNPs have been established in allotetraploid cotton. Development of putative 45,104 intraspecific and 17,954 interspecific SNP marker assays in cotton is valuable (Ashrafi et al., 2015). These advancements deliver a high throughput genotyping platform, basic and customary tool for genetic analysis of economically and agronomically significant traits associated with stress in cotton. A noteworthy proportion of the whole genome comprises of copy-number-variations (CNVs) rather than SNPs. Although, CNVs can be used for identification of phenotypic variations for complex traits (Abelson, 1998) as well as, tolerance to abiotic stress, which are normally not covered by SNPs. CNVs can alter gene regulation, dosage, and structure, in plant genomes and they can also affect genes associated with abiotic stress tolerance (Wang et al., 2017a). In cotton genome, CNV modifications in 989 genes have been detected that are linked with plant cell wall organization, translational regulation, and plant type (Zhang et al., 2015).

### 9.2. Transcriptomic profiling

Transcriptome profiling is a useful approach for extracting information from sequence data in order to learn about different gene activities and pathways. Various methods of transcriptome analysis like suppression subtractive hybridization, DNA microarray, and RNA-seq have been developed. RNA-seq has appeared as a powerful method for analyzing gene expression variations in plants that reflects the underlying underpinnings of salt stress responses (Mickelbart et al., 2015; Wang et al., 2017c).

Lately, the whole genome transcriptome was reported that offers information about expressed sequence tag (EST) assemblies of inbred line of Upland cotton, TM-1, and functions as a reference genome for all SNP studies that are RNA-sequence based (Ashrafi et al., 2015). Trancriptome libraries of *G. barbadense* for traits associated with stresses like salinity, heat, cold, and water stress, were also kept as a reference for identification of novel stress associated genes (Zhou et al., 2016). With the help of RNA-Seq analysis of wide-scale gene expression by next generation sequencing (NGS) technologies and tetraploid and diploid genome sequences, transcriptome analysis for salinity and other abiotic stresses in cotton has been performed (Zhang et al., 2018). The pattern of expression of differentially expressed genes (DEGs) showed the transcription of genes related to stress under environmental stress in somatic embryos in a comparative transcriptome analysis (Jin et al., 2014). Though, still some challenges remain for RNA-seq, like the requirement to process and store huge data sets and handling of the library construction.

In addition to genetic factors, the epigenetic based changes also control various gene functions in plants. Out of these, DNA methylation is the most common epigenetic signaling approach, as it plays a very important role in the evolution of morphological as well as physiological diversity in plants. In cotton, fiber development and other plant tissues have showed seasonal variation in modifications based on DNA methylation (Phillips, 2008; Suzuki and Bird, 2008; Jin et al., 2013; Osabe et al., 2014). Furthermore, DNA methylation plays an active role in development of ovule and fiber. Dependency of RNA-directed DNA methylation (RdDM) and CHH methylation has been detected for the stimulation of different genes in ovules. A type of DNA methylation i.e. chromomethylase 2 (CMT2), silences a few genes related to fiber development (Song et al., 2015). In recent times, epigenetic changes were used to modify 519 cotton genes in cultivated and wild species (Song et al., 2017).

One of the significant post transcriptional regulators of gene expression are small RNAs called microRNAs (miRNAs). miRNAs are small single stranded non coding RNAs ranging from 19 to 25 nucleotides in length (Carrington and Ambros, 2003). Cleavage and translational repression seems to be the predominant method of post transcriptional regulation in plants (Schwab et al., 2005; Brodersen et al., 2008). Recent studies found that some miRNA of some plants respond to stress situations and some miRNA targets are genes related to stress. This put forwards that miRNA plays a very significant role in response of plant to stress. Expression profiles of miRNA in response to salinity stress have been studied in *Arabidopsis thaliana* (Liu et al., 2008), *Oryza sativa* (Sunkar et al., 2008), *Zea mays* (Ding et al., 2009), *Gossypium hirsutum* (Yin et al., 2012), and *Caragana intermedia* (Zhu et al., 2013). Besides examining the effects of stress on miRNA, detection of miRNA targets is also very important. In cotton, though, some miRNA associated with salinity tolerance have been found to differ under salt stress conditions (Zhang et al., 2007; Pang et al., 2009; Ruan et al., 2009; Xue et al., 2013), as little information is available about their expression profiles in response to salinity stress and the role of these miRNA in adaptation to salt remains uncertain.

**Figure 4:**
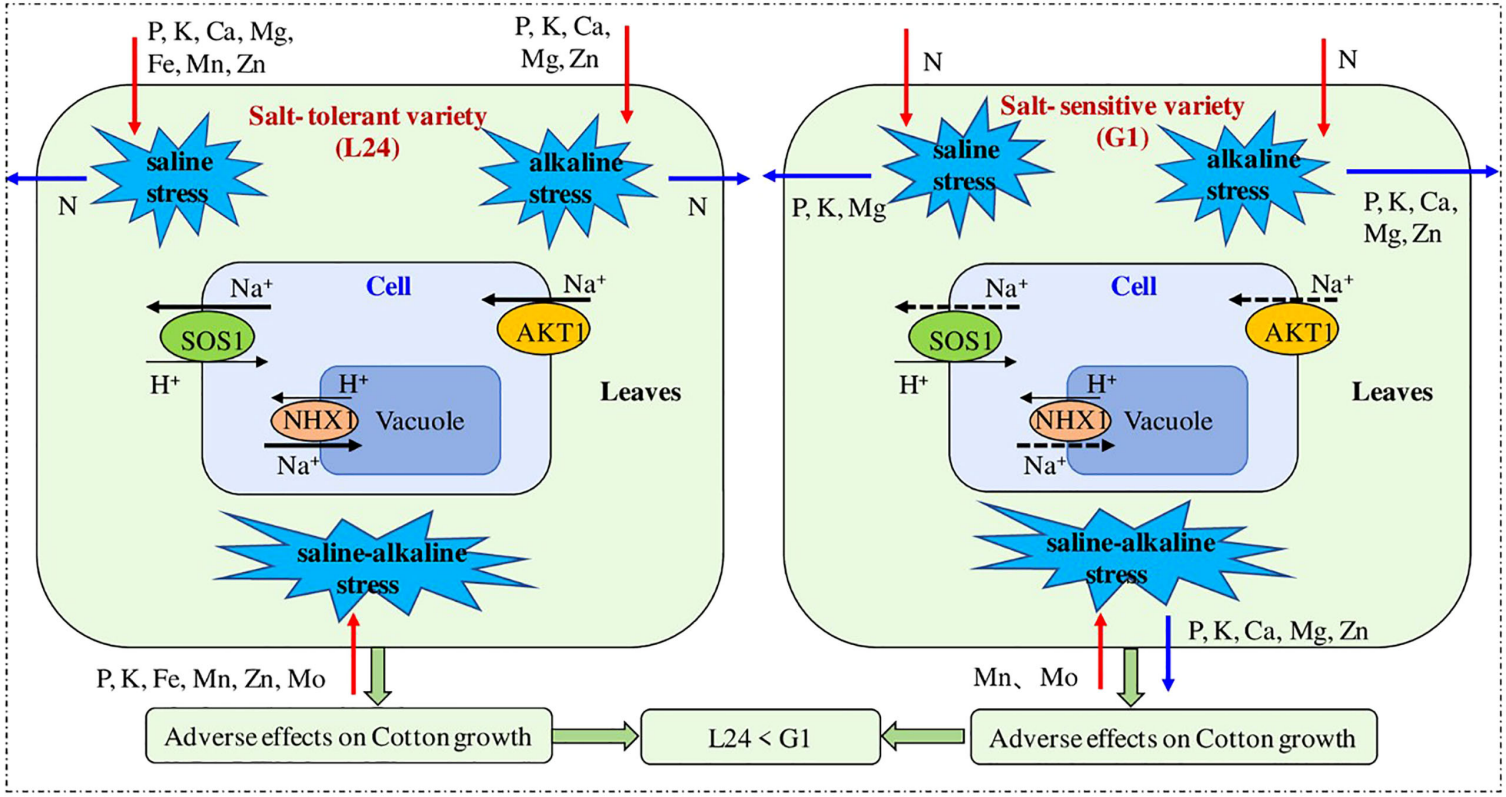
A study showing the comparison between a salt tolerant and salt sensitive cultivar in terms of response of ion content and gene expression (Sun et al., 2021) .

Few genes have been pinned down in cotton that express their selves under salt stress conditions e.g. DREB (Gao et al., 2009), MKK (Lu et al., 2013), GhMT3a (Xue et al., 2009), ERF (Johnson et al., 2003), and MPK (Zhang et al., 2011). A list of salt responsive genes in cotton is given in **Table 1**. A unique gene, GhNHX1, stimulates the tonoplast, Na+/H+ antiporter and also controls the defense mechanism of plant against salt stress. It was also observed that the mRNA level of GhNHX1 was greater in salt tolerant genetic material as compared to salt sensitive cultivars, signifying its role in salt tolerance (Wu et al., 2004). A study is shown in **Figure 4** in which a comparison is shown between salt sensitive and salt tolerant genotypes. The sensitive one showed greater inhibition in transport of K, P, and Mg and absorption of P.

**Table 1:** Functional genomics of salt responsive genes in Gossypium hirsutum L.

Genes	Function	References
GhNHX1	Tonoplast Na+/H+ antiporter	(Wu et al., 2004)
GhMT3a	Type 3 metallothionein protein	(Xue et al., 2009)
GhNAC4 GhNAC6	Encode NAC domain	(Meng et al., 2009)
GhDREB	Dehydration responsive element binding protein gene	(Gao et al., 2009)
GhERF6 GhERF2 GhERF3	ERF-encoding genes	(Jin et al., 2010)
GhDi19-1 GhDi19-2	Drought induced protein which is Cys2/His2 zinc-finger proteins	(Li et al., 2010)
GhMPK2	Mitogen-activated protein kinase gene	(Zhang et al., 2011)
GhNAC1 GhNAC6	Encode NAC domain	(Shah et al., 2013)
GhMKK1	Mitogen-activated protein kinase	(Lu et al., 2013)
GhSOD1	Superoxide dismutase	(Luo et al., 2013)
GhWRKY11 GhWRKY12 GhWRKY13 GhWRKY14 GhWRKY115 GhWRKY20 GhWRKY21 GhWRKY24 GhWRKY30 GhWRKY32 GhWRKY33 GhWRKY34	WRKY transcription factor	(Zhou et al., 2014)
GhWRKY39-1	WRKY transcription factor-encoding	(Shi et al., 2014)
GhWRKY39	WRKY transcription factor	(Shi et al., 2014)
GhAnn1	Annexin gene	(Zhang et al., 2015)
GhWRKY41	WRKY transcription factor-encoding	(Chu et al., 2015)
GhMAP3K40	Mitogen-activated protein kinase gene	(Chen et al., 2015)
GhCCL	Cold-circadian rhythm-RNA binding-like protein	(Dhandapani et al., 2015)
GhABF2	bZIP-encoding gene	(Liang et al., 2016)
GhWRKY25	WRKY transcription factor-encoding	(Liu et al., 2016)
GhTPS11	Trehalose-6-phosphate synthase	(Wang et al., 2016)
GhWRKY6	WRKY transcription factor	(Li et al., 2019)
GhZAT34, GhZAT79	genes of zinc finger proteins	(Rehman et al., 2021)

## 10. Genome-modification/transgenic approaches for salt tolerance improvement in cotton

It is now possible to transfer desirable traits or genes between plants and species to achieve a desired phenotype using genetic engineering and biotechnology. Transgenic techniques are valuable for enhancing plant abiotic stress tolerance following the discovery of many salt tolerance genes. The transgenic technique contributed in the development of salt tolerant transgenic plants as well as the identification of salt responsive genes.

Transcription factors play a very important role in response of plant to abiotic stresses. An example of such is GhABF2 which is a bZIP transcription factor gene. It was overexpressed in transgenic cotton plants which established salt tolerance through regulation of genes related to abscisic acid (ABA). These plants also yielded more compared to control non-transgenic plants, when exposed to salt stress (Liang et al., 2016).

Increased solute concentration in plant cell vacuoles (osmotic adjustment) is thought to be a key factor in improving salt tolerance in plants. Enhancing solute concentration increases Na^+^ accumulation in vacuoles while lowering the risk of Na^+^ toxicity in the cytoplasm, resulting in increased salt tolerance in transgenic plants. A transgenic plant of cotton was produced to over-express an vacuolar Na^+^/H^+^ antiporter of *Arabidopsis*, named AtNHX1 (He et al., 2005; He et al., 2007). More fiber yield and biomass was generated from cotton plants with AtNHX1 under high salt concentration. Increased sodium transfer into vacuoles occurs due to overexpression of both of the tonoplast Na^+^/H^+^ antiporters, resulting in increased vacuolar salt content and thus higher salinity tolerance.

Similarly, cotton plants overexpressing the transcription factor gene SNAC1 showed higher salinity tolerant with more vigorous rooting system and lesser transpiration rate as compared to non-transgenic plants. Another gene, *Arabidopsis* AtEDT1/HDG11 was overexpressed in transgenic cotton plants and these plants showed higher salt and drought tolerance, extensive root system, higher ROS scavenging enzymes activity, and more proline content in leaves (Liu et al., 2014; Yu et al., 2016). In comparison to non-transgenic cotton, overexpression of the *Arabidopsis* vacuolar Na^+^/H^+^ antiporter gene AtNHX1 in cotton led to higher drought and salt tolerance in greenhouse and field conditions, as well as higher fiber yield (Umezawa et al., 2006). Furthermore, a study co-overexpressed AtNHX1 and AVP1 genes in cotton and resulted in enhanced salinity and drought tolerance in transgenic plants (Shen et al., 2015).

Multiple salt stress-responsive proteins have been reported to modify miRNAs and affect alternative splicing processes in upland cotton. After 4 and 24 hours of salt stress, the results of the stringent screening of mRNA-seq and proteomic data between the salt-tolerant and salt-susceptible genotypes identified 63 and 85 candidate genes/proteins, respectively, relevant to salt tolerance (Peng et al., 2018). The CMO gene from *Atriplex hortensis* led to enhanced salt tolerance when overexpressed in cotton (Huijun et al., 2010). Under salt stress, several auxin-induced genes, such as GH3 (auxin-conjugating enzyme) and IAA, were up-regulated, whereas genes involved in auxin metabolism (synthesis and degradation) and auxin signal transduction, such as ARF (auxin response factor) and AFB2, were down-regulated. Many genes involved in brassinosteroid production and signal transduction, such as SMT2 (sterol methyltransferase 2) and BES1 (bri1-EMSsuppressor 1), were also shown to be considerably down-regulated during salt stress.

Some gibberellin signal transduction pathway genes, such as GAI (gibberellic acid insensitive), were drastically down-regulated. Some gibberellin production genes, such as gibberellin 2-oxidase and gibberellin 20-oxidase, were up or downregulated. Furthermore, it was found that three probe sets for CKX7 (cytokinin oxidase 7) were up-regulated (Yao et al., 2011).

The expression levels of most GhNHX genes are influenced by salt stress. GhNHX4A expression was significantly up-regulated by salt stress, precisely, in the endosomal group. GhNHX4A can partly restore the salt tolerance of the salt susceptible yeast mutant AXT3 in a yeast functional complementation test. Salt tolerance of cotton was reduced by silencing GhNHX4A expression, due to a rise in Na^+^ accumulation in stems and a drop in K^+^ accumulation in roots. The findings of this study could be used to further characterize the regulatory activities of NHX genes involved in cotton salt tolerance, particularly the endosomal-type GhNHX4A (Ma et al., 2020). In response to salt stress in cotton seedlings, differential hybridization was used to isolate GhNHX1 (Wu et al., 2004). The mRNA accumulation of GhNHX1 in cotton seedlings was highly enhanced by salt stress through Northern blot analysis. Function complementation was shown by GhNHX1 activity in a yeast tonoplast Na^+^/H^+^ antiporter mutant, thus providing that the antiporter is present in the vacuolar membrane. Tobacco plants that were transgenic with over-expressing GhNHX1 showed enhanced salinity tolerance as compared to non-transgenic plants (Wu et al., 2004). After stress treatments, the transgenic GhDREB wheat plants accumulated larger quantities of soluble sugar and chlorophyll in their leaves, indicating increased tolerance to severe salt stress (Gao et al., 2009).

### 10.1. CRISPR/Cas9

Even though transgenic techniques are promising, there are certain drawbacks, such as extended incubation durations and low transformation efficiency in case of cotton. The CRISPR/Cas9 system consists of two components: a clustered regularly interspaced short palindromic repeat (CRISPR) and an associated protein 9 (Cas9), both of which are found in bacteria (*Streptococcus pyogenes*). The CRISPR/Cas9 system discovery was a quantum leap, and it now acts as a multifunctional tool for gene editing in plants. It has been successfully employed for quick and targeted genome editing in a variety of model plant species (Belhaj et al., 2015). In *Arabidopsis*, the model plant, the first use of genome editing by CRISPR/Cas9 was made successful (Shan et al., 2013). Nevertheless, there have been few reports of CRISPR/Cas9 being used successfully in cotton. Multiple targeted genome editing in allotetraploid cotton was recently performed by targeting the GhCLA1 (chloroplast development gene) and GhARG (arginase discosoma red fluorescent protein 2) genes (Wang et al., 2017d). Cis-sequences are key regulatory elements of genes and stress responses in the promoter region of genes. It has also been reported that these cis-sequences play an important role in stress regulation (Liu et al., 2014). Targeting these cis-sequence locations using the CRISPR/Cas9 system has recently been proposed as a way to develop novel QTLs for analysis of genotypic and phenotypic variations that can help increase abiotic stress tolerance in plants like cotton (Zafar et al., 2020). Technical hurdles and low transformation efficiency, on the other hand, preclude its widespread usage in cotton.

Some other genetic engineering tools like Zinc finger nucleases (ZFNs), Transcription activator-like effector nucleases (TALENs), and Meganucleases (MGNs) are also being employed recently to modify plant genomes for coping with various abiotic stresses. ZFNs identify and bind to target DNA sites. This approach is extremely specific for targeted genetic engineering. TALENs have been utilized in plant research programs recently. They contain a non-specific DNA-cleaving nuclease attached to a DNA-binding domain which can be conveniently engineered for targeting any sequence (Chantre Nongpiur et al., 2016).

## 11. Conclusion and future prospects

Salinity is a grave challenge in guaranteeing food security around the world, with more than half of countries worldwide experiencing it. Salt induces ion toxicity, nutritional imbalances, and somatically induced water stress, all of which have negative consequences on plant growth, development, and crop establishment. Salt stress inhibits enzyme metabolic processes, limits nutrient uptake, and causes nutritional disorders, resulting in lower yield and fiber quality. Most of the cultivated accessions of key crop species are extremely or moderately sensitive to salt stress, and as a result, they do not perform well under saline conditions in the field. Though cotton is considered as moderately salt tolerant crop, still its critical developmental stages can be affected adversely by high salt concentrations. An inexpensive approach to manage salt stress in cotton could be the development of cotton cultivars tolerant to salt stress. Conveniently, genetic sources of salt tolerance have been identified in most agricultural species, and they can be used in breeding. However, the majority of germplasms with salt tolerance have been found in related wild or undomesticated species, making them unable to use in breeding programs due to inherent challenges. In cotton, mechanisms for displaying tolerance to various abiotic stresses appear to be interrelated and may share genetic elements.

Salt stress impairs biochemical, molecular, and physiological processes in cotton, resulting in stunted growth and development, as well as reduced photosynthetic rate, leaf and root size, biomass, yield and yield components, and fiber quality. As a result, variety of traits like these listed above, can be used to screen cotton for salt tolerance while cultivated in the field or in the greenhouse.

Because of the negative correlation between yield and abiotic stress tolerance, linkage drag between genes for desirable and undesirable traits remains a major barrier in improving cotton for abiotic stress resistance through direct selections. Nevertheless, with the use of molecular markers to map QTLs in bi-parental and multi-parental populations as well as wild populations, great progress has been achieved in understanding the genetic basis of salt tolerance. Molecular markers and mapping technologies have now made it possible to identify genes or QTLs of interest for complex traits like salt tolerance and transfer them from un-adapted genetic backgrounds into present cultivars more accurately with the help of the marker assisted selection (MAS) process. When using wild germplasm as genetic resources, one of the benefits of MAS is that it reduces the challenges associated with linkage drag. With this potential in mind, substantial efforts have been commenced, and several genes or QTLs related to salt tolerance in many plant species have been identified.

Many salt responsive genes have been identified in the last 10–15 years, with some of them being researched further with the help of transgenic techniques. Through genetic engineering, many genes linked with plant salt tolerance have been added to cotton to improve abiotic stress tolerance. It is important to note, however, that none of these genes have been used in commercial cotton breeding programs. Due to the interconnection of salt responses with various other aspects of plant growth, promising salt tolerance genes are frequently reported to have some unfavorable impacts on cotton growth *via* gene-silencing or overexpression.

The current state of transgenic cotton with improved salt tolerance is far behind what is required for commercial production. This is due to the fact that salt tolerance, so far, has not reached the level expected for cotton grown in salt affected fields, and also the strains of salt tolerant materials have rather poor agronomic performance. As a result, future research should concentrate on maximizing the extensive use of salt-tolerant genes, as well as successful transformation and development of new salt-tolerant cotton cultivars. Cotton improvement hinges on the development of transgenic cotton varieties with high salt tolerance and other well-integrated characteristics.

It is projected that more permanent inter-specific and intra-specific linkage mapping populations will be established with a variety of parent genomes for high resolution QTL mapping and repeated phenotyping for different stresses with the help of genome wide SNP markers. The QTLs having greater salt stress tolerance will be identified by MAS and tolerant genes will be introduced into high yielding cultivated species. Furthermore, to increase the consistency and range of cotton germplasm phenotyping for salt stress tolerance, rapid, reliable, and high throughput screening procedures valid on a broad scale should be developed.

## Author contributions

MKRK and ZM conceived and conceptualize the presented idea. ZM collected data, organize the contents in the form of review article and took the lead in writing the manuscript and overall finalization of the review article. TL and SN collected and organized literature for assigned topics. AD was in charge of overall direction and planning. BW and AD re-evaluated the study in the scientific sense before submission. SMUDK contributed in drafting and critical revision of the article. MKRK and BW overall organized and supervised the course of progress and took the responsibility of the study. All authors contributed to the article and approved the submitted version.

## Funding

We are highly thankful to the National Key R&D Program of China (2021YFE0101200), Pakistan Science Foundation PSF/CRP/18^th^Protocol (07), the, Higher Education Commission (HEC) (HEC Indigenous Scholarship/Phase II-Batch V), Pakistan, International Foundation for Science (IFS) Sweden and COMSTECH, Islamabad, Pakistan, (IFS-I-1-C-6500-1), and Ministry of Planning Development and Special Initiatives Pakistan, PSDP Project No. 829 for providing funding for this research program on salt tolerance of cotton germplasm.

## Acknowledgments

We are indebted to give appreciation to the Pakistan Institute of Engineering and Applied Sciences (PIEAS), Islamabad and the Department of Biological Sciences, Nuclear Institute for Agriculture and Biology (NIAB), Faisalabad, for providing an umbrella for the research program. We highly appreciate the Central Cotton Research Institute (CCRI) Multan for providing the valuable cotton germplasm. We express the profound sense of reverence to the entire research team, friends, and any other person who contributed; we have deep gratitude for you so much.

## Conflict of interest

The authors declare that the research was conducted in the absence of any commercial or financial relationships that could be construed as a potential conflict of interest.

## Publisher’s note

All claims expressed in this article are solely those of the authors and do not necessarily represent those of their affiliated organizations, or those of the publisher, the editors and the reviewers. Any product that may be evaluated in this article, or claim that may be made by its manufacturer, is not guaranteed or endorsed by the publisher.
